# Impaired renal function before kidney procurement has a deleterious impact on allograft survival in very old deceased kidney donors

**DOI:** 10.1038/s41598-021-91843-7

**Published:** 2021-06-09

**Authors:** Mehdi Maanaoui, François Provôt, Sébastien Bouyé, Arnaud Lionet, Rémi Lenain, Victor Fages, Marie Frimat, Céline Lebas, François Glowacki, Marc Hazzan

**Affiliations:** 1 Nephrology Department, University of Lille, CHU Lille, 59000 Lille, France; 2 Univ. Lille, Inserm, CHU Lille, Institut Pasteur Lille, U1190 - EGID, 59000 Lille, France; 3 Urology Department, University of Lille, CHU Lille, 59000 Lille, France; 4 University of Lille, INSERM UMR995, 59000 Lille, France; 5 University of Lille, EA4483, 59000 Lille, France; 6 Service de Nephrologie, Hôpital Huriez, CHRU de Lille, 59037 Lille, France

**Keywords:** Nephrology, Kidney

## Abstract

As the use of elderly kidney donors for transplantation is increasing with time, there is a need to understand which factors impact on their prognosis. No data exist on the impact of an impaired renal function (IRF) in such population. 116 kidney recipients from deceased kidney donors over 70 years were included from 2005 to 2015 in a single-center retrospective study. IRF before organ procurement was defined as a serum creatinine above 1.0 mg/dl or a transient episode of oligo-anuria. Mean ages for donors and recipients were respectively 74.8 ± 3.5 and 66.7 ± 8.0. Graft survival censored for death at 5 years was of 77%. Using a multivariate analysis by Cox model, the only predictor of graft loss present in the donor was IRF before organ procurement (HR 4.2 CI95[1.8–9.7]). IRF was also associated with significant lower estimated glomerular filtration rates up to 1 year post-transplantation. By contrast, KDPI score (median of 98 [96–100]), was not associated with the risk of graft failure. Then, IRF before kidney procurement may define a risk subgroup among very-old deceased kidney donors, in whom pre-implantatory biopsies, dual kidney transplantation or calcineurin inhibitor-free immunosuppressive regimen could help to improve outcomes.

## Introduction

In order to face the organ shortage crisis, the proportion of kidneys from deceased donors older than 70 years has significantly increased in the past few years. However, the use of elderly kidney donors varies between countries. For instance, in 2015 only 4.9% of deceased kidney donors were older than 65 years in the U.S^1^, compared to 35% in France^2^. The rate of discarded kidneys, which is almost twice as high in the US than in France, partly explains this difference^3^. Thus, it is crucial to identify specific prognostic factors related to these marginal kidneys in order to determine subgroups at risk which may benefit from protective strategies. Moreover, a recent analysis of disparities between France and the US revealed that a more aggressive policy of acceptance, especially in elderly donors, may reduce drastically the rate of discarded kidneys^4^.

Until 2014 in the U.S, grafts were classified as extended criteria donors (ECD) kidneys or standard criteria donors (SCD) kidneys^5^. However, this binary classification did not take into consideration other comorbidities apart from age, hypertension, serum creatinine and stroke, which are frequently observed in older donors. In 2014, the Organ Procurement and Transplantation Network and United Network for Organ Sharing (OPTN/UNOS) introduced a continuous score in the U.S allocation system, namely the Kidney Donor Profile Index (KDPI), based on 10 donor-factors to better estimate the quality of the graft^6^. The KDPI was built to be used in tandem with the estimated post-transplantation survival (EPTS) score for recipients in order to attribute the best kidneys to the best recipients, moving from an equity to a usefulness paradigm^7^. However, the KDPI did not reduce the discard rate of marginal kidneys. Indeed, analysis of the OPTN/UNOS register revealed that up to 62% of kidneys with a KDPI above 90% are not transplanted^8^. It is noteworthy that the KDPI of a donor older than 70 years, without any comorbidities, is higher than 80%.

The present study focused on identifying risk factors for graft loss, in a population of recipients transplanted from deceased donors older than 70 years, in order to refine the prognosis of transplantation. Considering that kidney aging is associated with altered regenerative abilities^9^, we tested if an impaired renal function (IRF) prior to organ removal could impact long-term allograft outcomes. Indeed, donor renal function has never been evaluated in these old donors. The impact of donor renal function on kidney transplantation outcomes is difficult to assess, as controversy exists in the literature regarding the way to evaluate it. For instance, recent large-scaled studies did not demonstrate deleterious effects of donor acute kidney injury (AKI) on long-term outcomes, using the standard definition of KDIGO^10–12^. In the particular case of very-old donor kidneys, where the functional reserve may be decreased because of aging, we hypothesized that the combination of the peak serum creatinine value and the urine output, defining IRF, could be associated with graft outcomes.

Therefore, we retrospectively analyzed graft outcomes from deceased kidney donors older than 70 years according to the presence of IRF before organ procurement.

## Results

### Donors’ and recipients’ baseline characteristics

From 01/01/2005 to 31/12/2015, 116/1461 (8%) recipients received a kidney from a deceased donor older than 70 years. Median follow-up was 34 months (17–52).

All donors’ demographic characteristics are summarized in Table 1. Briefly, mean age was 74.8 ± 3.5 and most of them died from cerebrovascular events (75.9%). Mean KDPI was 97.1 ± 3.5.

**Table 1:** Baseline donors’ characteristics with comparison according to a peak serum creatinine over 1.0 mg/dl and/or a transient oligo-anuria in the donor.

	Overall cohort (n = 116)	NoIRF group (n = 85)	IRF group (n = 31)	*p*-value
Male, n (%)	50 (43.1)	32 (37.6)	18 (58.1)	0.08
**Age, mean ± SD**	74.8 ± 3.5	74.9 ± 3.2	74.6 ± 4.2	0.72
70–74, n (%)	54 (46.5)	38 (44.7)	16 (51.6)	
75–79, n (%)	49 (42)	38 (44.7)	11 (35.4)	
> 80, n (%)	13 (11.5)	9 (10.6)	4 (13)	
BMI, mean ± SD	27.6 ± 5.4	26.9 ± 5.0	29.5 ± 6.0	0.04
**Donor source, n (%)**				0.27
DBD	115 (99.1)	85 (100)	30 (96.8)	
DCD	1 (0.9)	0 (0)	1 (3.2)	
**Cause of death, n (%)**				0.62
Stroke	88 (75.9)	66 (77.6)	22 (71.0)	
Trauma	23 (19.8)	17 (20.0)	6 (19.4)	
Anoxia	5 (4.3)	2 (2.4)	3 (9.6)	
**Comorbidities, n (%)**				
Diabetes	14 (12.1)	7 (8.2)	7 (22.6)	0.05
Hypertension	72 (62.1)	54 (63.5)	18 (58.1)	0.75
Stroke	15 (12.9)	12 (14.1)	3 (9.7)	0.76
Tobacco consumption	12 (10.3)	8 (9.4)	4 (12.9)	0.73
Coronary heart disease	18 (15.5)	16 (18.5)	2 (6.5)	0.15
Chronic heart failure	10 (8.6)	9 (10.6)	1 (3.2)	0.29
KDPI, median (IQR)	98 (96–100)	98 (95–100)	99 (98–100)	0.01
**Before organ removal**				
Recovered cardiac arrest, n (%)	13 (13)	5 (6.0)	10 (32.3)	0.001
Use of pressor amines, n (%)	110 (94.8)	80 (94.1)	30 (96.8)	1.00
Transient oligo-anuria, n (%)	8 (6.9)	0	8 (25.8)	0.001
Serum urea (g/l), mean ± SD	0.34 ± 0.27	0.30 ± 0.27	0.45 ± 0.23	0.007
Serum creatinine (mg/dl), median (IQR)	0.80 (0.66–1.02)	0.72 (0.60–0.81)	1.22 (1.07–1.40)	0.001
Proteinuria over 1 g/L, n (%)	24 (23.8)	17 (22.7)	7 (26.9)	1.00
Renal arteries calcification, n (%)	45 (51.6)	35 (51.5)	13 (51.2)	1.00
Perfusion machine, n (%)	44 (37.9)	33 (38.8)	11 (35.5)	0.91
CIT, minutes, mean ± SD	1037 ± 285	1029 ± 290	1057 ± 273	0.64
WIT, minutes, mean ± SD	108 ± 50.5	105 ± 47.8	118 ± 56.9	0.27

For all tests, a p-value < 0.05 was considered as significant.

*BMI* Body Mass Index, *DBD* brain deceased donor, *DCD* donor after cardiac death, *CIT* cold ischemia time, *IQR* interquartile range, *KDPI* Kidney Donor Profile Index, *N/A* non appliable, *IRF* Impaired Renal Function, *SCr* serum creatinine, *WIT* warm ischemia time.

31 donors (26.7%) presented with impaired renal function (IRF group) defined as oligo-anuria (25.8 of them versus 0% in the control group, p < 0.001) and/or SCr > 1 mg/dl at the time of procurement (1.22 IQR[1.07–1.40] versus 0.72 IQR[0.60–0.81], p < 0.001). The 1.0 mg/dl peak serum creatinine value cut-off corresponded to the fourth quartile of the distribution and was data-driven as it was associated with a significant lower graft survival when compared to other quartiles (Supplementary Fig. 1). When compared to the 85 remaining donors without renal failure (NoIRF group) they presented a higher body mass index (29.5 ± 6 versus 26.9 ± 5, p = 0.04), a more frequent diabetic history (22.6 versus 8.2%, p = 0.05) and their KDPI was slightly higher (98.2 ± 2.4 versus 96.7 ± 3.8, p = 0.014). 32.3% of them had recovered from a cardiac arrest before procurement (versus 6% in the control group, p < 0.001).

Demographic characteristics of the recipients are summarized in Table 2. Mean age was 66.7 ± 8.0. 14.7% of them had already benefited from previous kidney transplantation and 31% presented with preformed HLA antibodies. Median waiting time on dialysis was 31.5 (18–47.5) months. The recipients in the IRF group presented with a more frequent history of stroke (19.4% versus 3.5%, p = 0.011) and peripheral arteritis (19.4% versus 3.5%, p = 0.011). However, the Charlson comorbidity index was similar in both groups (5.7 ± 1.5 versus 5.3 ± 1.6, NS).

**Table 2:** Baseline recipients’ characteristics with comparison according to a peak serum creatinine over 1.0 mg/dl and/or a transient oligo-anuria in the donor.

	Overall (n = 116)	NoIRF group (n = 85)	IRF group (n = 31)	*p*-value
Male, n (%)	71 (61.2)	57 (67.1)	14 (45.2)	0.05
Age, mean ± SD	66.7 ± 8.0	66.3 ± 8.6	68.0 ± 5.8	0.23
BMI, mean ± SD	26.2 ± 3.9	26.0 ± 3.5	26.5 ± 4.9	0.51
**Cause of ESRD, n (%)**				0.74
Diabetes	16 (13.8)	10 (11.8)	6 (19.4)	
Glomerular disease	33 (28.4)	22 (25.9)	11 (35.5)	
Interstitial	10 (8.62)	9 (10.6)	1 (3.2)	
Vascular	16 (13.8)	13 (15.3)	3 (9.7)	
Cystic disease	24 (20.7)	18 (21.2)	6 (19.4)	
Undetermined	13 (11.2)	10 (11.8)	3 (9.7)	
Other urologic disease	4 (3.45)	3 (3.5)	1 (3.2)	
**Comorbidities, n (%)**				
Diabetes	29 (25.0)	19 (22.4)	10 (32.3)	0.40
Hypertension	75 (64.7)	54 (63.5)	21 (67.7)	0.84
Stroke	9 (7.8)	3 (3.5)	6 (19.4)	0.01
Peripheral arteritis	9 (7.8)	3 (3.5)	6 (19.4)	0.01
Tobacco consumption	14 (12.1)	13 (15.3)	1 (3.2)	0.11
Obesity	15 (12.9)	8 (9.4)	7 (22.6)	0.11
Coronary heart disease	11 (9.5)	7 (8.2)	4 (12.9)	0.48
Arrythmia	25 (21.6)	18 (21.2)	7 (22.6)	1.00
chronic heart failure	11 (9.5)	7 (8.2)	4 (12.9)	0.48
COPD	9 (7.76)	5 (5.9)	4 (12.9)	0.25
Charlson comorbidity index, mean ± SD	5.2 ± 1.6	5.3 ± 1.6	5.7 ± 1.5	0.34
Waiting time on dialysis, median (IQR)	31.5 (18.0–47.5)	34.0 (19.0–49.0)	26.0 (16.0–46.0)	0.47
Previous transplantation, %	17 (14.7)	15 (17.6)	2 (6.5)	0.23
Sensitization, %	40 (34.5)	32 (37.6)	8 (25.8)	0.33
total HLA mismatch, mean ± SD	5.1 ± 1.8	5.1 ± 1.7	5.1 ± 2.0	0.89

For all tests, a p-value < 0.05 was considered as significant.

*BMI* Body Mass Index, *COPD* chronic obstructive pulmonary disease, *ESRD* end-stage renal disease, *IQR* interquartile range, *IRF* Impaired Renal Function, *SCr* serum creatinine.

### Post-transplantation outcomes

As shown in Table 3, the prevalence of DGF, surgical complications, acute rejection and infections was not significantly different between the 2 groups.

**Table 3:** Post-transplantation outcomes with comparison according to a peak serum creatinine over 1.0 mg/dl and/or a transient oligo-anuria in the donor.

	Overall (n = 116)	NoIRF group (n = 85)	IRF group (n = 31)	*p*-value
Length of stay in hospital, days, mean ± SD	17.1 ± 9.9	15.9 ± 9.1	20.2 ± 11.6	0.07
PGNF, n (%)	8 (6.9)	4 (4.7)	4 (12.9)	0.21
DGF, n (%)	29 (25.2)	19 (22.6)	10 (32.3)	0.41
**eGFR (MDRD), mean ± SD**				
Day 15	26.2 ± 12.9	27.5 ± 13.4	22.5 ± 11.0	0.08
M1	31.1 ± 12.7	32.7 ± 12.7	26.7 ± 12.4	0.03
M3	36.4 ± 12.8	37.6 ± 12.5	32.2 ± 13.0	0.06
M6	39.0 ± 13.9	40.7 ± 14.1	32.9 ± 12.0	0.02
M12	37.2 ± 13.9	38.9 ± 13.9	30.3 ± 12.3	0.02
Acute ABMR, n (%)	8 (6.9)	7 (8.2)	1 (3.2)	1.00
Acute cellular rejection, n (%)	14 (12.2)	10 (11.9)	4 (12.9)	1.00
Chronic ABMR, n (%)	2 (1.7)	2 (2.4)	0 (0)	1.00
Chronic cellular rejection, n (%)	4 (3.5)	2 (2.4)	2 (6.7)	0.28
**Surgical complications, n (%)**				
Ureteral stenosis	11 (9.6)	6 (7.1)	5 (16.1)	0.16
Haematoma	10 (8.7)	6 (7.1)	4 (12.9)	0.46
Vesicoureteral reflux	5 (4.3)	4 (4.8)	1 (3.2)	1.00
Urinoma	6 (5.2)	5 (4.8)	2 (6.5)	0.66
Lymphocele	1 (0.9)	1 (1.2)	0 (0)	1.00
Renal artery stenosis	6 (5.2)	5 (5.9)	1 (3.2)	1.00
**Infectious diseases, n (%)**				
Bacterial infection	60 (52.2)	39 (46.4)	21 (67.7)	0.07
CMV disease	14 (12.1)	15 (17.6)	6 (19.4)	1.00
BK polyomavirus nephropathy	3 (2.6)	1 (1.2)	2 (6.5)	0.17
Aspergillosis	3 (2.6)	2 (2.4)	1 (3.2)	1.00
Pneumocystosis	8 (6.9)	6 (7.1)	2 (6.5)	1.00
Cancer, n (%)	24 (20.9	16 (19.0)	8 (25.8)	0.59

*ABMR* antibody-mediated rejection, *CMV* Cytomegalovirus, *DGF* delayed graft functioning, *eGFR* estimated glomerular filtration rate, *IRF* Impaired Renal Function, *PGNF* primary graft nonfunctioning.

However, IRF was associated with a significant lower eGFR from month 1 (26.7 ± 12.4 versus 32.7 ± 12.7 ml/min/1.73 m^2^, p = 0.03) up to month 12 post transplantation (30.3 ± 12.3 versus 38.9 ± 13.9 ml/min/1.73 m^2^, p = 0.02).

Overall death-censored graft survival rates were respectively 91%, 86% and 77% at year 1, 3 and 5 post-transplant. Death-uncensored graft survival rates were respectively 83%, 73% and 59% at year 1, 3, and 5 post-transplant.

**Figure 1:**
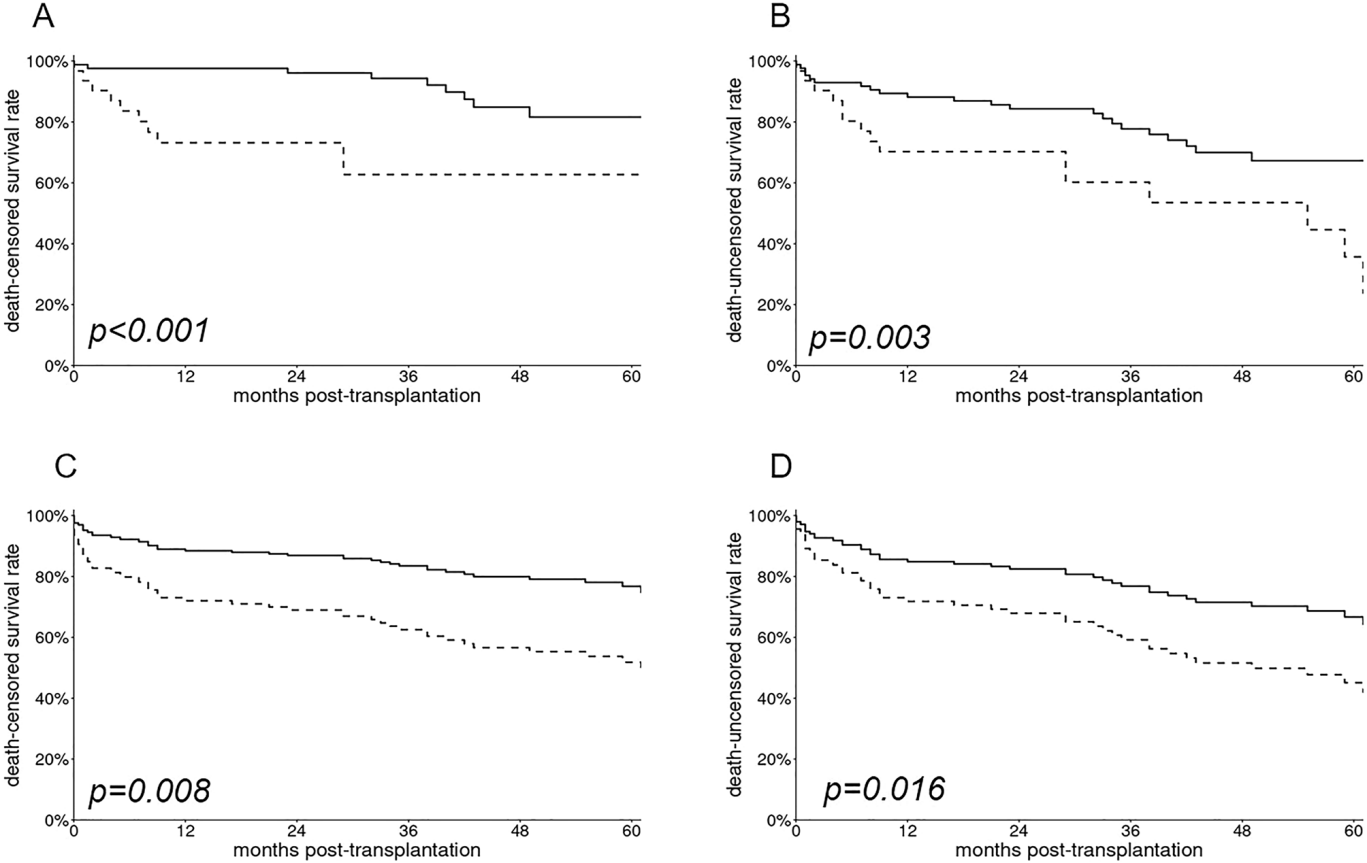
Graft survival rates: non-adjusted survival rates death-censored (**A**) and death-uncensored (**B**) and adjusted graft survival rate, death-censored (**C**) and death-uncensored (**D**). (**C**) Adjustment for post-transplant hematoma, acute rejection, BK virus nephropathy and BMI. (**D**) Adjustment for post-transplant hematoma and urinoma, acute rejection and BK virus nephropathy. Bold line = donor impaired renal function group. Dashed line = no donor impaired renal function group. Donor renal failure was defined as a donor with a serum creatinine over 1.0 mg/dl or with a transient oligo-anuria before organ procurement. p-values for non-adjusted curves were defined according to the log-rank test. p-values for adjusted curves were defined according to the Cox model analyses.

IRF was associated with lower death-censored (Fig. 1A, p < 0.001) or death-uncensored (Fig. 1B, p = 0.003) non-adjusted graft survival rates.

### Risk factors for graft loss

Cox regression models were built (Tables 4, 5) to identify independent risk factors for graft loss in the overall population. Among recipient’s factors, univariate analysis revealed that post-transplant hematoma and urinoma, acute rejection and BK virus nephropathy were significantly associated with a higher risk of graft failure. BMI above 30 kg/m^2^ was also a risk factor of death-censored graft loss. Among donor’s related parameters, IRF was the only significant risk factor, both in death-censored and death-uncensored univariate analyses. In multivariate analysis, it remained significantly associated with a higher risk of graft loss (HR 4.0 [1.4–11.3] and 2.3 [1.2–4.4] for death-censored and death-uncensored multivariate models, respectively) and lower adjusted death-censored (Fig. 1C, p = 0.008) and death-uncensored (Fig. 1D, p = 0.016) graft survival rates.

**Table 4:** Univariate Cox regression model for risk factors of death-censored and death-uncensored graft loss.

	Death-censored graft survival		Death-uncensored graft survival	
	Univariate	*p-*value	Univariate	*p-*value
	HR [95% CI]		HR [95% CI]	
**Recipient**				
Age	0.99 [0.94–1.04]	0.611	1.00 [0.97–1.04]	0.813
Sex (male)	1.93 [0.75–4.98]	0.176	1.51 [0.78–2.93]	0.227
BMI (> 30 kg/m^2^)	3.58 [1.39–9.19]	0.008	1.68 [0.77–3.70]	0.195
Waiting time on dialysis > 30 months	0.81 [0.34–1.97]	0.627	1.06 [0.57–1.96]	0.853
Previous transplantation	2.10 [0.75–5.85]	0.155	1.53 [0.70–3.33]	0.289
Sensitization	0.51 [0.17–1.52]	0.229	0.63 [0.30–1.33]	0.228
DGF	1.74 [0.72–4.22]	0.219	1.18 [0.60–2.31]	0.626
Total HLA mismatch	1.10 [0.17–0.52]	0.482	1.09 [0.90–1.31]	0.386
Charlson comorbidity index	1.10 [0.84–1.44]	0.507	1.09 [0.90–1.32]	0.655
Post-transplant hematoma	3.22 [0.92–11.28]	0.067	3.48 [1.43–8.46]	0.003
Post-transplant urinoma	6.27 [1.77–22.19]	0.004	3.85 [1.34–11.05]	0.017
Acute rejection	5.82 [1.94–17.42]	0.002	2.76 [1.15–6.61]	0.023
BK virus nephropathy	10.24 [1.79–58.69]	0.009	6.47 [2.32–18.03]	< 0.001
**Donor**				
Age	1.01 [0.87–1.16]	0.931	0.95 [0.85–1.06]	0.362
Sex (male)	1.26 [0.54–2.94]	0.587	1.14 [0.61–2.11]	0.682
BMI (kg/m^2^)	1.05 [0.95–1.13]	0.234	1.03 [0.97–1.09]	0.385
Diabetes	2.07 [0.68–6.30]	0.199	1.41 [0.59–3.38]	0.444
Hypertension	0.82 [0.34–1.98]	0.658	0.82 [0.43–1.57]	0.556
Recovered cardiac arrest	0.98 [0.22–4.27]	0.978	1.55 [0.64–3.72]	0.329
KDPI	0.99 [0.87–1.11]	0.835	0.99 [0.88–1.02]	0.167
Oligo-anuria	5.35 [1.74–16.43]	0.003	3.97 [1.64–9.63]	0.002
SCr > 1 mg/dl	3.92 [1.61–9.53]	0.003	2.41 [1.26–4.63]	0.008
IRF: SCr > 1 mg/dl and/or oligo-anuria	4.20 [1.80–9.70]	0.001	2.50 [1.30–4.80]	0.005
Cold ischemia time > 17 h	0.85 [0.32–2.26]	0.749	0.88 [0.42–1.86]	0.853
Hypothermic perfusion machine	1.22 [0.44–3.42]	0.706	0.81 [0.37–1.79]	0.610

All variables with a p-value under 0.2 in univariate analyses were introduced in the multivariate models. For all tests, a p-value < 0.05 was considered as significant.

*BMI* Body Mass Index, *DGF* delayed graft-function, *SCr* serum creatinine.

**Table 5:** Multivariable Cox regression model for risk factors of death-censored and death-uncensored graft loss in the overall population.

	Death-censored graft survival		Death-uncensored graft survival	
	Multivariate	*p-*value	Multivariate	*p-*value
	HR [95% CI]		HR [95% CI]	
**Recipient**				
Post-transplant hematoma	6.1 [2.1–17.9]	0.001	3.8 [1.9–7.6]	0.001
Post-transplant urinoma	–	–	3.7 [1.5–8.9]	0.004
Acute rejection	13.6 [4.3–42.9]	0.001	3.2 [1.4–7.4]	0.005
BK virus nephropathy	11.5 [1.3–97.4]	0.025	5.4 [1.4–17.8]	0.005
BMI (> 30 kg/m^2^)	6.1 [2.4–15.6]	0.001	–	–
**Donor**				
IRF: SCr > 1 mg/dl or oligo-anuria	4.0 [1.4–11.3]	0.008	2.3 [1.2–4.4]	0.016

All variables with a p-value under 0.2 in univariate analyses were introduced in the multivariate models. For all tests, a p-value < 0.05 was considered as significant.

*BMI* Body Mass Index, *SCr* serum creatinine.

Other donor and recipients related variables such as cardiovascular comorbidities, post-transplant cardiovascular events, infections (see Supplementary Table 1), were also not significant.

## Discussion

Several studies have reported the outcomes of renal transplant recipients who received a kidney from a deceased donor older than 70 years (Table 6)^13–20^. However, none of these studies have analyzed the impact of donor IRF before kidney procurement. In our study, we provide for the first-time evidence that IRF has a deleterious impact on long-term outcomes for donors older than 70 years old. Indeed we found that a peak serum creatinine above 1.0 mg/dl and/or an oligo-anuria episode before organ procurement is associated with a lower eGFR up to 1 year post transplantation and impairs graft survival, both in death-censored and death-uncensored analyses, after adjustment for confounding factors. These results may reflect a lower tissue repair capacity after ischemia–reperfusion^9^, due to kidney aging, which would account for the persistent altered renal function at 1 year. We used the serum creatinine peak instead of the standard classification of AKI^11^ or the final serum creatinine for several reasons^21^. It remains difficult to define renal function in deceased-donors, as their baseline serum creatinine is rarely available and the changes in the serum creatinine values during organ procurement may depends of hemodynamic parameters as well as haemodilution. Furthermore the impact of donor AKI on kidney transplantation outcomes is still controversial although recent large-scaled studies did not demonstrate deleterious effects^10^. The serum creatinine peak can reflect the renal function reserve which can be reduced in old donors and could be a relevant parameter in this population. Indeed an increased last serum creatinine in such donors leads frequently to kidney discard. The present study is limited by the sample size, and a larger cohort would be required to explore the impact of different serum creatinine cutoffs, although large observational studies would also be limited due to the reluctance to accept very-old donors’ kidney with a last serum creatinine above 1.5 mg/dl. Overall death-censored and death-uncensored 5-year graft survival rates were 77% and 59% respectively in line with previous reports^13–20^ (Table 6). These results may be considered as acceptable, since the median age of recipients was 66 (63–72) years, close to a so-called “old-for-old” allocation. Indeed Lloveras et al. showed that kidney recipients from donors older than 65 years, had a better prognosis than patients remaining on the waiting list, after matching for sex, age, primary renal disease, time on dialysis and cardiovascular comorbidities^22^. However, in the present study, when the donor presented with IRF before organ procurement, 5-year graft survival rates decreased to 55.3% and 49.8% for death-censored and death-uncensored analyses, respectively. This poor prognosis could jeopardize the benefits from transplantation, even in old recipients.

**Table 6:** Overview of published cohorts on donors aged more than 70 or 75 years old.

First author, year of publication	Donors minimum age	Number of patients	5-year GS-DC, %	5-year GS-DNC, %	Graft survival—risk factors
Chavalitdhamrong, 2008^13^	70	601	67	44	Ethnicity^a^, previous transplantation, time on dialysis, diabetes^a^
Collini, 2009^14^	75	38	N/A	N/A	N/A
Foss, 2009^15^	75	54	83	59	N/A
Gavela, 2009^16^	70	53	N/A	70	N/A
Galeano, 2010^17^	70	70	70	N/A	HLA-DR mismatch, DGF
Gallinat, 2011^18^	75	52	N/A	53	Dual kidney transplantation
Machado, 2012^19^	70	60	80	77	N/A
Marconi, 2012^20^	70	82	N/A	N/A	DGF, acute rejection

*N/A* non appliable, *DGF* delayed graft functioning, *GS-DC* graft survival censored for death, *GS-DNC* graft survival non censored for death.

^a^Related to the recipient.

Unlike impaired renal function before procurement, KDPI failed to discriminate the “bad” and “good” grafts in these very old donors. Indeed KDPI was very high (97.1 ± 3.5), higher than 90% in most donors (97.4%). This could explain why KDPI was not associated with transplantation outcomes in this specific population, and may not be an accurate marker to assess the graft quality in older donors. Data from other European countries^23–25^ validated the use of KDPI to evaluate the graft prognosis. However KDPI is strongly correlated to age^26^. In line with our results, Dahmen et al. found that KDPI was higher that 90% when the donor age was over 70. Considering the kidney discard rates due to a high-KDPI in the US, we assume that most kidneys in the present study would have been discarded although Massie et al. showed a survival benefit in transplantations with high-KDPI kidney donors compared to patients remaining on the waiting list^27^. In the present study, recipients who received a kidney from an old donor without IRF had death-censored and death-uncensored 5-year survival graft rates of 79.1% and 70.2%, respectively, despite a KDPI at 96.7 ± 3.8. This confirms that such kidneys are worthy to be transplanted, if carefully selected, especially in older recipients.

The aim of our study was to provide easy tools to better assess the risk when using very-old kidney donors. The present results suggest that a peak serum creatinine level above 1.0 mg/dl could lead to better investigate the quality of the graft. In this context a pre-implantatory biopsy would help to assess the presence of acute tubular injuries or chronic lesions related to an underlying CKD, as suggested by the pre-implantatory Remuzzi score^28^, in order to refine acceptance and utilization of these kidneys (i.e. single or dual transplantation). Moreover this strategy can significantly reduce the discard rate without worsening the outcomes^3,29^. This is however dependent on high-quality standards to perform the biopsies and on dedicated analyses made by trained pathologists^30,31^. Considering only the donors presenting with peak serum creatinine above 1.0 mg/dl may rationalize resources and facilitate this strategy in routine practice. Other strategies to improve the prognosis of these very-old kidneys are the use of the perfusion machine and calcineurin inhibitor-free regimen. The use of hypothermic perfusion machine is indeed associated with better outcomes for ECD-recipients, both for the risk of DGF and graft loss^32^. However our data collection was not designed to study the effect of hypothermic perfusion machine. In the present study we did not find any association with graft survival in univariate analysis, although it seemed to be protective considering the risk of DGF (data not shown). Calcineurin inhibitor-free regimen may also be an alternative to improve long term results. Nevertheless, to date no study found a benefit of these strategies on graft survival^33–35^. Indeed the 7-year results of the BENEFIT-EXT clinical trial revealed better glomerular filtration rates in ECD-recipients^36^ but did not significantly reduce the graft loss rate.

This study carries several limitations. First, data were retrospectively collected, which conveys a risk of information loss. Data regarding the exposure and the definition of IRF may be partly biased. Indeed, oligo-anuria is determined and defined according to the French Registry, and the number of serum creatinine measurements per donor may influence the characterization of the donor status. Second, this is a small-sized single center cohort. Other variables, such as donor age, diabetes, hypertension, and others, may be not significant because of a lack of power. Recipients with IRF also presented more vascular comorbidities (background of stroke or peripheral arteritis) which worsen the long-term outcomes. Due to the sample size of the present study, we were not able to stratify on other variables, such as donor vascular comorbidities or cause of death, which would result in a very small number of events in each strata. Third, the European population included in the present study may significantly differs from the U.S population. Indeed, donor ethnicity could not be included in this analysis, due to French ethical issues^37^ and it seems likely that the proportion of African-Americans may affect post-transplantation outcomes in the U.S^38^. Ethnicity accounts for a significant part of the calculation of KDPI in the US system^6^, which might bias our conclusions. Thus, comparison regarding donors, discard rates and transplant outcomes in the US system and European countries should be interpreted with caution, and requires further investigation in wider cohorts. Ultimately, our results suggest that the peak serum creatinine could help to better assess the risk of graft failure in very-old donors where KDPI is systematically above 90%. Markers of kidney injuries (i.e. a peak SCr over 1 mg/dl and/or an oligo-anuria episode before organ procurement) should warn of the risk of poor transplantation outcomes. However, our findings cannot provide evidence to discard these grafts. First we did not assess the benefit to be transplanted with these marginal kidneys compared to stay longer on the waiting list, expecting another graft proposal. Second, we did not analyze the discarded kidneys characteristics. Thus our study does not intend to affect the decision-making process to accept or refuse these grafts. It only suggests that, in very old donors, KDPI does not provide a sufficient discrimination level to guide the physician’s choice.

To conclude, in the current context of organ shortage where very-old donors remain an important pool of kidneys, impaired renal function before kidney procurement could lead to histological evaluation in order to refine acceptance and allocation.

## Patients and methods

### Data source and ethical statement

This study was performed according to the Declaration of Helsinki and the Declaration of Istanbul. All data were collected from the CRISTAL database (French Biomedical Agency, which rules the allocation system in France) and from the recipients’ medical files. No organs were procured from prisoners. As the French Biomedical Agency regulates the allocation system in France, every organ was allocated by the Agency and transplanted in Lille, France (Centre Hospitalier Régional, Lille). Ethical committee was bypassed, according to French laws and the local institutional review board (Centre Hospitalier Régional Universitaire de Lille), as the study was monocentric and observational. Informed consent was obtained from all subjects. No subjects under 18 were involved in the study. Patients and laboratory data were analyzed anonymously and registered in respect with the French data protection registry (Commission Nationale de l’Informatique et des Libertés, i.e. CNIL), referenced #DEC16-235.

### Study design

This is a retrospective monocentric study performed at the Lille University Hospital, France. All consecutive adult recipients who were transplanted between the 1st of January 2005 and the 31st of December 2015, with a kidney from deceased kidney donors older than 70 years were included. All of them received an induction therapy consisting in either basiliximab (20 mg at day 0 and day 4) for non-sensitized recipient older than 55 years or thymoglobulin (1.25 mg/kg from day 0 to day 3) for recipient younger than 55 years or presenting with HLA immunization. Maintenance immunosuppression associated tacrolimus, mycophenolate mofetil and steroids. Early steroid withdrawal (day 7) was performed in non-sensitized recipients. Valganciclovir was given during the first 6 months post transplantation in Cytomegalovirus (CMV) seronegative patients who received a CMV seropositive kidney. Prophylaxis for *Pneumocystis jirovecii* (trimethoprim-sulfamethoxazole) was given during the first 3–6 months post transplantation.

The following donors’ parameters were collected: age, sex, weight, height, HLA antigens, comorbidities (diabetes, hypertension, cardiovascular diseases, heart failure, and tobacco consumption), type of donor [brain deceased donor (DBD) or donor deceased after cardiac arrest (DCD)], cause of death, KDPI score, hemodynamic data (cardiac arrest, use of pressor amines) and renal function (urine output, serum creatinine, serum urea, and proteinuria) before organ procurement. Cold and warm ischemia times as well as the conservation modality (hypothermic perfusion machine (HPM) or static cold storage) were also registered.

The following recipients’ baseline parameters were collected: age, sex, weight, height, HLA antigens, comorbidities [diabetes, hypertension, coronary artery diseases, stroke, peripheral arteritis, arrhythmia, heart failure, tobacco consumption, chronic obstructive pulmonary disease (COPD), and cirrhosis], cause of end stage renal disease (ESRD), time on dialysis, time on waiting list, Charlson comorbidity index, dual kidney transplantation, previous transplantation, HLA sensitization. After transplantation, main complications (immediate post-transplantation hematoma, urinoma, or lymphocele, infections, and cardiovascular events), estimated glomerular filtration rate (eGFR using MDRD formula) at day 15, months 1, 3, 6, 12, and acute rejection episodes were registered. Delayed graft function (DGF) was defined as the need for dialysis during the first week post transplantation. Primary graft non-function (PGNF) was defined as failure of the graft to function the first 3 months after transplantation.

The KDPI score for each donor was retrospectively calculated using the OPTN calculator (https://optn.transplant.hrsa.gov/resources/allocation-calculators/kdpi-calculator/), except for ethnicity which is not available in the CRISTAL database.

### Exposure

In order to avoid collinearity, donor variables “oligo-anuria” and “serum creatinine > 1.0 mg/dl” were tested separately in the univariate analysis and then pooled in one variable IRF in the multivariate analysis.

Donor IRF before procurement was defined as following:

- a peak serum creatinine above 1.0 mg/dl. The threshold of 1.0 mg/dl represents the fourth quartile of serum creatinine peak in this very-old donor cohort.
- and/or a transient episode of oligo-anuria in intensive care unit before the organ procurement defined by KDIGO stage I (< 0.5 ml/kg/h for 6 h), according to data available in the French CRISTAL Registry.

### Statistical analysis

Qualitative variables were expressed in number and percentage. Quantitative variables were expressed in means and standard deviations or in median and interquartile according to their distribution estimated by the Shapiro–Wilk test.

Qualitative variables were compared by a chi-2 test. A student t-test or a Mann–Whitney test, when appropriate, was used to compare quantitative variables. Actuarial survivals were depicted with the Kaplan–Meier method and compared by the log-rank test. A Cox model was used to identify factors associated with graft survival, censored or not for death. All the variables with a p-value under 0.2 in univariate analysis were introduced in the multivariate models. Acute rejection and BK virus infection were analyzed as time dependent variables. A stepwise regression using a backward elimination was performed to obtain the final multivariate model.

For all tests a p-value < 0.05 was considered as significant. The statistical analysis was performed with R software (R Development Core Team (2008). R: A language and environment for statistical computing. R Foundation for Statistical Computing, Vienna, Austria).

## Supplementary Information

Supplementary Information.
