# Supplementary material for: Impaired renal function before kidney procurement has a deleterious impact on allograft survival in very old deceased kidney donors
Source: Sci Rep. 2021 Jun 9;11:12226. doi: 10.1038/s41598-021-91843-7 (PMC8190122; doi:10.1038/s41598-021-91843-7)
Supplement: Supplementary file 1 — Supplementary Information. [file 41598_2021_91843_MOESM1_ESM.pdf]

Impaired renal function before kidney procurement have a deleterious impact on allograft survival in very old deceased kidney donors

Mehdi Maanaoui[1,2], MD, François Provôt[1], MD, Sébastien Bouyé[3], MD, Arnaud Lionet[1], MD, Rémi Lenain[1], MD, Victor Fages[1], MD, Marie Frimat[1,4], MD, PhD, Céline Lebas[1], MD, François Glowacki[1,5], MD, PhD, Marc Hazzan[1], MD, PhD.

1. University of Lille, CHU Lille, Nephrology Department, F-59000, Lille, France
2. University of Lille, INSERM U1190, Translational Research for Diabetes, Lille, France
3. University of Lille, CHU Lille, Urology Department, F-59000, Lille, France
4. University of Lille, INSERM UMR995, F-59000, Lille, France
5. University of Lille, EA4483, F-59000, Lille, France

Corresponding author contact information:

Mehdi MAANAOUI, Service de Nephrologie, Hôpital Huriez, CHRU de Lille, 59037 Lille, France. Tel: +33 631924327 Email: mehdi.maanaoui@gmail.com

SUPPLEMENTAL TABLE 1 List of covariates included into the Cox model analyses.

	Covariates
Donor	Age, sex, BMI, diabetes, hypertension, cardiovascular diseases, heart failure, tobacco consumption, cause of death, KDPI score, recovered cardiac arrest, use of pressor amines, transient anuria, serum creatinine, serum urea, proteinuria, cold and warm ischemia time, use of hypothermic perfusion machine.
Recipient	Age, sex, diabetes, hypertension, coronary artery diseases, stroke, peripheral arteritis, arrhythmia, heart failure, tobacco consumption, chronic obstructive pulmonary disease, cirrhosis, cause of end stage renal failure, previous transplantation, EPTS score, HLA sensitization, HLA mismatches, waiting time dialysis and on the waiting list, Charlson comorbidity index, previous transplantation.
Transplantation	Immediate post-transplant hematoma, urinoma or seroma, infections, cardiovascular events, BK polyomavirus nephropathy, biopsy-proven acute rejection.

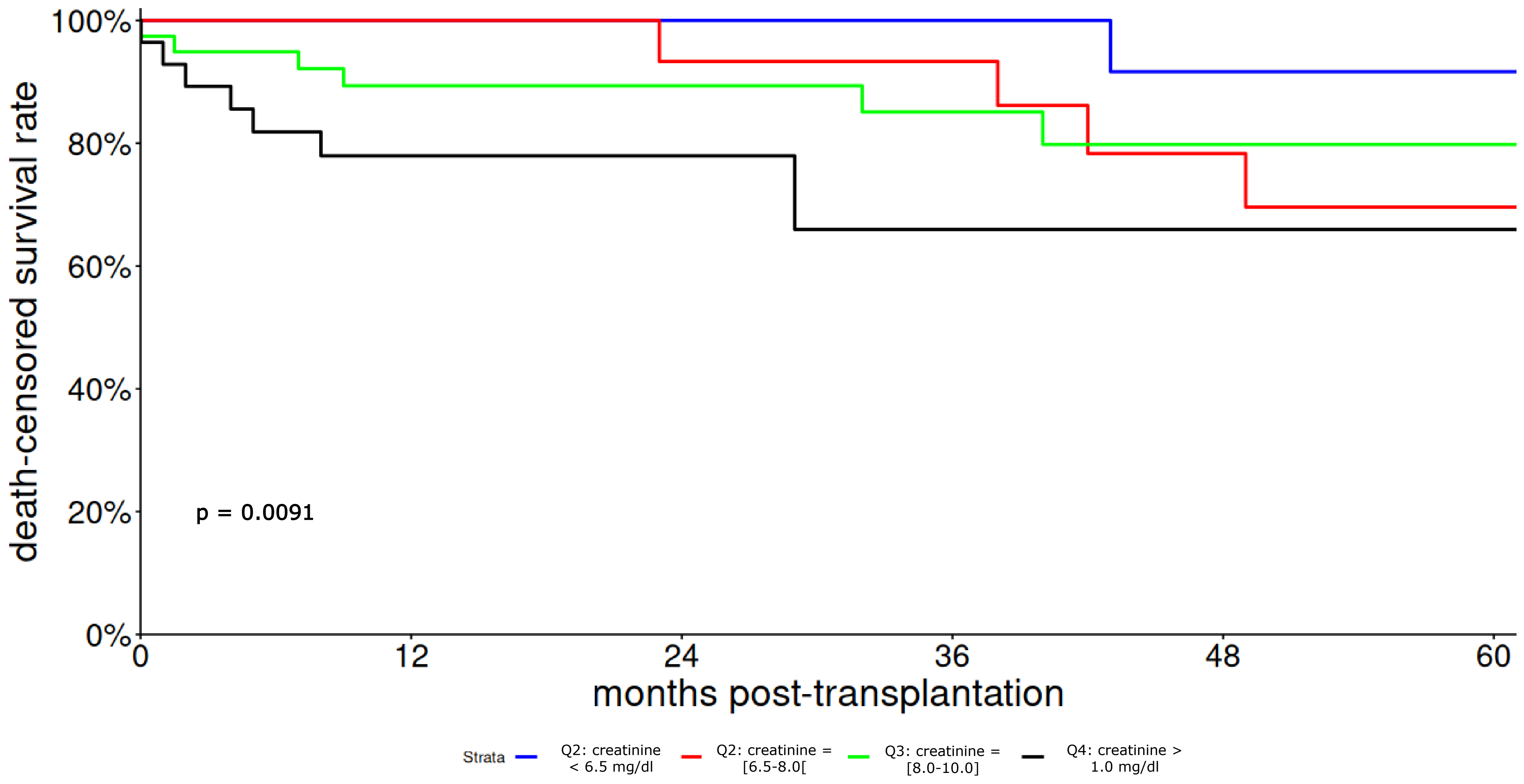

Supplemental Figure 1. Death-censored graft survival rates according to peak serum creatinine quartiles. p-values were defined according to the log-rank test.
